# “What if It’s not Just an Item of Clothing?” – A Narrative Review and Synthesis of the White Coat in the Context of Aged Care

**DOI:** 10.5334/pb.1138

**Published:** 2022-02-23

**Authors:** Coline Crutzen, Stéphane Adam

**Affiliations:** 1 Psychology of Aging Unit, University of Liège (B63C), Liège, Belgium

**Keywords:** white coat, ageing, white coat effect, enclothed cognition, uniform, nursing home

## Abstract

Although increasingly disputed, the white coat uniform is ubiquitous in geriatric care, which may reflect a phenomenon called medicalisation of ageing. This narrative review is the first attempt at integrating several theoretical approaches, such as the “white coat effect” and “enclothed cognition”, in order to gain a comprehensive understanding of the use of this clothing item. Based on extensive empirical evidence, we will examine the consequences of wearing a uniform, not only on patients (in this case, older patients) and healthcare professionals, but also on their relationship. The white coat has powerful symbolic functions for healthcare professionals and is still preferred by older adults. However, the negative repercussions of wearing a uniform require us to question its use, particularly in environments where older persons live, such as nursing homes.

## Introduction

Increasing life expectancy in industrial society is reflected in the growing number of older people. This rapid increase has caused the need for developing facilities dedicated to this specific population, but at the same time has led to ageing being seen as a “problem”, a “burden” that society must bear (Officer et al., 2020). Analysing the content of over 150,000 stories about ageing (from newspapers, books, and magazines), Ng and Chow (2020) observed a linear increase in ageism over the past 210 years. Ageism refers to “the process of systematic stereotyping and discrimination against people because they are old” (Butler, 1969). The way we talk about ageing has never been as negative as it is today. One predictor of this development is the medicalisation of ageing, which manifests by using physical health descriptors when talking about ageing or older persons. Instead of considering ageing as a normal, highly individualised, multidimensional process of growth involving maintenance and decline (Rattan, 2014), we tend to reduce it to a single story of medical decline (Lucchetti et al., 2017). As a result, what should be considered as part of the natural course of life (e.g., physical and cognitive ageing, menopause, or death) comes to be regarded as a medical problem requiring clinical intervention for its treatment and elimination (van Dijk et al., 2019; Calasanti & King, 2020). Ageism and the medicalisation of ageing, therefore, mutually feed into each other since ageism contributes to the pathologising of ageing, which, in turn, encourages the development of negative stereotypes about ageing.

The trend towards the medicalisation of ageing took off in the second half of the 20th century with the emergence of geriatrics (Gilleard & Higgs, 2013). While this phenomenon has had a positive impact on access to care, it has also encouraged society to view ageing as a pathological or abnormal event (Calasanti & King, 2020). Several examples help illustrate this phenomenon. Ataseven Bulun and Ünal (2019) found that the most common ageing-related topics discussed in current print media are prevention and beauty loss (e.g., how can one stay young and beautiful). The authors explain that one of the purposes of medicalisation of the ageing body is to market anti-ageing remedies. The emergence of «mild cognitive impairment», or MCI, in the DSM-5 (Diagnostic and Statistical Manual of Mental Disorders) also reflects the increasing medicalisation of cognitive and brain ageing (Van der Linden & Juillerat, 2014).

Nowadays, it seems that ageing is considered a pathological phenomenon. Hence, the factors characteristic of medicalisation (i.e., medical language, diagnostic labels, medical interventions, medical environments, and clothing) accompany this process even though it is actually developmental. It is therefore not surprising to note that, even in places where older adults live (nursing homes, their own homes), healthcare professionals put on the white coat for both care and daily tasks.

In the current context of the medicalisation of ageing, we would like to focus on one clothing item in particular, the white coat, a well-known symbol of care, and on the impact of its use. In this narrative review, we will discuss the consequences of wearing a white coat for the patient (the “white coat effect” theory), for the health professional (the “enclothed cognition” theory), and on the relationship between members of these two groups. To date, no synthesis of these different theoretical frameworks has been made. However, it is through this global approach that we can challenge our practices in the care and support of older adults.

## The white coat and our preferences

The white coat uniform was introduced into the medical field in the 19th century (Pearson et al., 2001) under the influence of the army (military) and the Church. Although its design has evolved over the years, this uniform has come to occupy a prominent position in our society and is certainly one of the most representative symbols of care (Brandt, 2003).

On the one hand, the white coat is associated with professionalism, competence, and knowledge, which can arouse a sense of trust and safety for the patient (Thomas et al., 2010; Chang et al., 2011; Gherardi et al., 2009; Tiang et al., 2017; Zahrina et al., 2018). On the other hand, the uniform is associated with authority and power (Gooden et al., 2001; Rehman et al., 2005; Tiang et al., 2017) which can induce fear and submission (McLean & Naidoo, 2006). A health professional wearing a white coat could therefore be perceived, ambivalently, as competent but cold (Nome Eikhom et al., 2006). This ambiguity would explain why, during surveys, patients admit they prefer white coats to civilian clothes, while simultaneously finding the white-coated professionals less friendly and approachable (Chang et al., 2011; Barrett & Booth, 1994).

A systematic review by Petrilli et al. (2015), which included 30 studies involving 11,533 patients from 14 different countries, refined the understanding of preferences by taking into account variables such as patients’ age. This review indicates that in 60% of cases, adult patients prefer the professional to wear formal attire or a white coat. This preference is much more pronounced in older patients. According to Anvik (1990), this generational difference can be explained by the fact that younger people have grown up in societies where self-determination and participation have become part of democratic trends. According to the author, this hypothesis can be understood in the context of the abandonment of the white coat by healthcare professionals (doctors), in countries that have done so as a symbolic manifestation of their desire to no longer be associated with power in favour of a more democratic doctor-patient relationship. Preferences may also differ depending on the context of care. Indeed, if the rapid identification of professionals by their uniforms is justified in certain cases such as emergencies, it appears less important in familiar, non-emergency or prolonged situations (e.g.: a visit to the family doctor, institutionalisation). In such situations, patients’ preference for the white coat is less pronounced or even absent (Long et al., 2017; McLean & Naidoo, 2006).

The white coat is thus part of the preferences consciously expressed by patients, particularly older ones. However, at a more unconscious level, it can negatively impact the physiological, cognitive, or emotional states of older individuals.

## Effects of the white coat on the patient (“White coat effect”)

### White Coat Effect (WCE): physiological repercussions

The white coat effect (WCE) was first observed in 1896 by an Italian physician, Riva-Rocci, who noted a rise in patients’ blood pressure during a doctor’s visit. However, the WCE has only been studied quantitatively since 1983 (Cobos et al., 2015). While there is no agreed-upon definition, the WCE can be described as the phenomenon of raised blood pressure that occurs during a clinical visit, that later dissipates (Pickering et al., 2002). It is indeed common to observe higher blood pressure in the adult population when measured in a clinical setting, manually (i.e., by a health professional), compared to when measured at home by a monitoring device (electronic device that does not require the presence of a professional). This phenomenon can be explained by: the involvement of a white-coated health professional, as well as the medical environment (Adiyaman et al., 2015). As there are different methods to assess the white coat effect (for a synthesis, see Pickering et al., 2002), it is currently difficult to determine whether the increase in blood pressure is due to the presence of a health professional and/or the wearing of a white coat by the professional and/or being in a medical environment.

Blood pressure can rise merely because a doctor (or a healthcare professional wearing a white coat) enters a room (Parati et al., 1998). In a meta-analysis of 26 studies and a total of 5,407 participants, Bo et al. (2021) found that the prevalence of the WCE (specifically: difference in blood pressure measured at the doctor’s office versus at home) declines by half when the measurement at the doctor’s office is performed using an electronic device without the presence of a healthcare professional. The prevalence drops from 14% to 7%, which confirms the effect a professional wearing a white coat has. However, in an experimental study specifically designed to distinguish between “the physician effect” and “the hospital effect”, Adiyaman et al. (2015) found that the WCE was more likely to be attributed to the hospital environment. In this study, 3 blood pressure measurements were performed and compared: (a) home blood pressure reading (with a monitoring device), (b) an office blood pressure reading (in a hospital) in the absence of a physician, (c) an office blood pressure reading with a physician present (the monitoring device used by the physician). By comparing measurements (a) and (b), researchers calculated “the hospital effect” (the environment effect). “The physician effect” was determined by comparing measurements (b) and (c).

The WCE tends to be more pronounced in older patients (Reddy et al., 2014; Manios et al., 2008). An analysis of data from 257 participants in the Jackson Heart Study, for example, compared clinic and ambulatory (in-home) blood pressure measured among American adults with hypertension aged < 60 vs. ≥ 60 years old. The difference between clinic and in-home blood pressure was, on average, higher among those aged 60 or older compared to those under 60 (12 mm Hg higher vs. 8 mm Hg higher) (Tanner et al., 2016). The WCE is generally greater when blood pressure is measured by a physician than by a nurse (La Batide-Alanore et al., 2000; Myers et al., 1995), as well as when measured by an experienced physician versus a young physician (La Batide-Alanore et al., 2000). This suggests that the magnitude of the WCE partly depends on the status of the professional. It could also depend on the interactions between the patient and the professional taking the measurement (Pickering et al., 2002). As suggested by Cobos et al. (2015), a healthcare professional with better communicational skills and a greater level of empathy reduces patient anxiety, which would reduce the likelihood of experiencing “white coat hypertension” (WCH).

Although the mechanisms underlying the WCE are still poorly understood, hypotheses point towards psychological and emotional factors such as stress and anxiety (Cobos et al., 2015; Nuredini et al., 2020). According to Grassi et al. (1999), this is an alert reaction similar to the defence reactions observed in animals when faced with emotional stressors. This state of alertness activates the sympathetic nervous system and leads to vasoconstriction and an increase in blood pressure. According to Ogedegbe et al. (2008), the WCE can be explained by a classical conditioning phenomenon that works as follows: the fear of being diagnosed with hypertension (or any other diagnosis) constitutes an unconditional stimulus that generates an (unconditional) anxiety response, causing an increase in blood pressure. Following one or more associations with the unconditioned stimulus, the initially neutral stimuli, such as the doctor’s office and the white coat, would become conditioned stimuli. These could then generate by themselves a conditioned anxiety response leading to an increase in blood pressure. Bloomfield & Park (2017) also conclude that the WCE is a frequently observed neuroendocrine reflex that is conditioned by the anticipation of a blood pressure measurement and the fear of what that measurement might reveal about any future diseases.

The concept of the WCE differs slightly from “white coat hypertension”, which refers to a group of people with hypertension in a clinical setting but whose blood pressure is normal in other settings (Pickering et al., 2002). However, these two concepts are not easily distinguishable because there is no clear consensus about them in scientific literature (Celis & Fagard, 2004). Some authors suggest that the WCE refers to the event itself (i.e., the increase in blood pressure), while the WCH refers to the diagnosis given to people who experience this effect (Bloomfield & Park, 2017). For others, these concepts are both diagnoses that are part of the “white coat syndrome”. (Pioli et al., 2018; Cuspidi et al., 2018). More recently, Cohen et al. (2019) has suggested that WCH should be used for people who receive medication, and WCE for those who do not. The number of people who suffer from hypertension in this type of environment (i.e., the WCH group) is relatively high since it affects between 10% and 50% of the population (Gorostidi et al., 2015). For these individuals, the risk of developing a cardiovascular disease is higher than that of normotensive people (Pioli et al., 2018). A meta-analysis by Cohen et al. (2019), which included 27 studies with a total of 25,786 participants, concluded that untreated subjects with WCH had a higher risk of cardiovascular events, cardiovascular mortality, and all-cause mortality than normotensive control subjects.

### White Coat Effect (WCE): cognitive impact

Some environments are likely to have a negative impact on cognitive performance, more specifically on the memory of older adults. In a study by Sindi et al. (2013), researchers found higher levels of cortisol (stress hormone) and poorer memory performance in older adults when assessed in an adverse and stressful context. This unfavourable context was an unfamiliar environment, where older subjects performed an explicitly presented word recall task during the afternoon with a professional who was significantly younger than them. This testing environment is considered “unfavourable” for older adults because it promotes a “stereotype threat” effect (Barber, 2020). In other words, the different characteristics of this environment (such as being evaluated by a young person) would increase the fear that older individuals have of confirming the stereotype that is widespread in our society, namely that memory declines with age (Follenfant & Atzeni, 2020). When an individual is confronted with a situation where they risks confirming a stereotype, this generates a state of anxiety that can negatively impact their performance (Marquet et al., 2016).

Similarly, a medical environment is likely to create a “threat of diagnosis”, i.e. a fear of being diagnosed and of being ill (Mazerolle et al., 2020; Scholl & Sabat, 2008). This threat would provoke a state of stress in an individual, which would negatively impact their cognitive performance (Suhr & Gunstad, 2002; Suhr & Gunstad, 2005).

This has been observed by Schlemmer and Desrichard (2018), who suggest that the WCE may extend beyond the phenomenon of increased blood pressure and can also impact the cognitive sphere. In their study, 27 older subjects (aged 64 to 74) were randomly assigned to two experimental conditions. Under control condition, the experimenter administered memory tests in a neutral room. She wore civilian clothing and introduced herself as a psychologist. Under experimental condition, memory tests were carried out in a medical setting, the experimenter wore a white coat and presented herself as a neuropsychologist. The results showed an interaction effect between the environment (medical vs. neutral) and the memory self-efficacy of the older person (measured through the MSE – Memory Self-Efficacy Questionnaire). These results highlighted that the medical environment impairs the memory performance of older people with a low memory self-efficacy, but boosts the memory performance of older adults with a high memory self-efficacy. In other words, the memory performance of older individuals who have a low perception of their memory capacity is likely to be impaired simply due to being assessed in a medical environment by a person wearing a white coat. However, it is unclear from this study which variable (the room, the experimenter’s clothing, the experimenter’s status, or all three) influences memory performance. To the best of our knowledge, no other study has tested the specific effect of wearing the white coat on cognition in older people.

Thus, the WCE is a phenomenon specific to the clinical context, influenced as much by physical factors (e.g., age) as by psychosocial factors (e.g., interaction with the healthcare professional) and socio-environmental factors (e.g., medical environment). Through a conditioning effect or a threat of diagnosis, the white coat and the medical environment, associated with the concept of «disease», are likely to impair the health of an older person, particularly pertaining to their blood pressure or memory capacity. This phenomenon therefore highlights the deleterious impact that a physical and social environment dominated by healthcare can have on older individuals.

## Effects of the white coat on the professional (“Enclothed cognition”)

The impact of the white coat is not limited to patients, since recent studies suggest that it could also impact the person wearing it. Studies on this topic are part of the research field of “enclothed cognition”. According to the central idea of this theory, developed by Adam and Galinsky (2012), wearing particular clothing items leads us to embody that clothing and their symbolic meaning. Thus, wearing a white coat may not only influence the people we interact with, but also our own psychological processes. Clothing or accessories that have symbolic meaning are associated with abstract concepts (e.g., the clown’s red nose is associated with the abstract concepts of laughter or comedy). When these symbolic clothes are worn, the associated abstract concepts are activated, which can impact the psychological state and behaviour of the wearer (e.g., if one wears a red nose, the activation of the associated abstract concept causes one to become more inclined to be funny and willing to make people laugh). Adam and Galinsky (2012) specify that “enclothed cognition” implies the coexistence of two independent factors, (a) the association of a symbolic value with the clothing, and (b) the physical experience of wearing the clothing. This second condition distinguishes “enclothed cognition” from the material priming effect, which can be defined as the phenomenon where the mere exposure to a physical object promotes behaviours that are consistent with the symbolic value associated with that object.

The initial experimental study carried out by Adam and Galinsky (2012) was able to demonstrate the white coat impact on the attentional processes of the wearer. Since the white coat is the typical clothing of doctors and scientists, the authors hypothesised that it would be symbolically associated with being careful and prudent. This association was confirmed by an online survey, which was completed by 38 subjects. The authors then discovered, using a Stroop Test (a test of inhibition and attention), that subjects who wore white coats during the test (n = 30) made approximately 50% fewer errors than as subjects dressed in civilian clothes (n = 30), during the interference part of the test. This finding would indicate a higher level of selective attention for participants in white coats.

The purpose of the second experiment, carried out by the same authors, was to test the specific effect of each of the two cumulative factors at the basis of the “enclothed cognition” theory, namely (a) the symbolic value of the item of clothing, and (b) the fact of wearing (not just looking at) the item of clothing. The symbolic value of the item of clothing was manipulated by presenting the coat either as belonging to a doctor (condition 1) or a painter (condition 2). To confirm that “enclothed cognition” goes beyond a simple material priming effect and that it is essential that the item of clothing be worn, a third condition was added, in which the subject was visually exposed to the white coat (presented as a doctor’s coat) but did not wear it (condition 3). The attentional dimension (sustained attention) was tested through a comparative visual search task (i.e., finding differences between pairs of photographs). 74 subjects (mean age: 19.85 years old) were randomised to the three conditions. Consistent with the authors’ hypotheses, subjects wearing a white coat presented as belonging to a doctor (condition 1) found more errors in the pictures compared to subjects in the other two groups (conditions 2 and 3), indicating a higher level of sustained attention. A third experiment with an identical design, involving 99 randomly assigned subjects, confirmed these results.

If the white coat can impact attentional processes, a recent study showed that it can also influence self-perception (Jones et al., 2019). Among elementary school students (n = 216), simply wearing a white (scientist’s) lab coat during science class significantly increased the feeling of being recognised by others as a scientist. By wearing this uniform, students also felt more competent and more often aspired to pursue a career in science. Note, however, that these effects were only observed among students who had initially perceived themselves as having low proficiency in this subject.

The studies more relevant to our research are those conducted by López-Pérez et al. (2016). Beyond the influence that the white coat can have on cognitive processes, these authors were able to demonstrate the influence this item of clothing can have on the emotions and behaviours of the wearer. A healthcare professional’s white coat (presented as a nurse’s coat in this particular case) is associated with the concepts of compassion, care, and help (see pilot study by López-Pérez et al., 2016). The authors speculated that wearing a healthcare professional’s coat would make us more empathetic and more likely to exhibit pro-social behaviours (i.e., helping behaviours). To test this hypothesis, three groups of 50 adult subjects (total n = 150) were asked to respond to an empathy scale (the Empathic Response Scale, Batson et al., 1987) and to take part in a computer game called the Zurich Prosocial Game (Leiberg et al., 2011), which aims to measure (virtually) the subject’s readiness to express helping behaviour. In the first group, subjects wore a nurse’s coat. In the second group, the subjects wore an identical coat, but this time it was described as belonging to a surface technician. Finally, in the third group, subjects looked at the nurse’s coat but did not wear it. Based on this first study, the authors established that subjects wearing a nurse’s coat (Group 1) showed significantly greater empathic concern and were more likely to provide assistance (88% for Group 1 vs. 16% and 50% for Group 2 and 3, respectively). Furthermore, this (virtual) help was provided more quickly by the first group.

In a second study, the authors proposed a real (not virtual) measure of pro-social behaviour. Adult subjects in all three groups (total n = 100) were given the opportunity to assist a (fictional) student presented as being in financial distress. To help this student, subjects could choose whether to engage in volunteer administrative assistance (lasting one to five hours) that allowed the student to obtain financial support. The results of this second study were consistent with those of the first study, as the authors found that the condition in which subjects provided the most help was that of wearing a nurse’s gown (64% for Group 1 vs. 44% and 32% for Groups 2 and 3, respectively).

This research shows that, in accordance with the “enclothed cognition” hypothesis, wearing a healthcare professional’s coat influences the emotional and behavioural responses of the wearer. Thus, by simply wearing this item of clothing, we are more likely to help those around us. This benevolent help can be beneficial in many cases. However, when help is provided when it is not necessary (over-helping), it can have damaging effects. It has been shown that when an older person is helped (physically) to perform a task that they are capable of doing, their ability of performing the task diminishes, along with their confidence (Avorn & Langer, 1982). However, due to the ageism rooted in our society and our institutions, older adults are mostly perceived as incompetent, fragile, and vulnerable (Cuddy et al., 2005; Fernández-Ballesteros et al., 2020). These stereotypes are even more ingrained among healthcare professionals (reference removed from the blinded version). By perceiving older adults as incompetent and identifying themselves as helpful healthcare professionals by wearing a white coat (“enclothed cognition” effect), they risk providing excessive help, generating the opposite effect of what is expected, namely diminished performance and a loss of autonomy for the older person.

Excessive help that we tend to provide to older adults, which stems from our paternalistic attitudes (Vale et al., 2020; Sánchez-Izquierdo et al., 2019), can be reinforced by wearing a uniform. Moreover, healthcare professionals who stop wearing their white coat for a few months (or even more), tend to discover that patients or residents become less demanding and more independent (Sparrow, 1991; Bailly et al., 2020). Instead of passively complying, patients or residents become more active and engaged (Chu et al., 2020). The uniform would thus maintain the role of “passive recipient of care” in patients or residents.

## Effects of the white coat on the patient-proffesional relationship

If the white coat somehow helps shape the patient and the professional, it is also through this uniform, a symbol of power and authority, that the dominant position of the professional can be displayed (Chu et al., 2020; McLean & Naidoo, 2006). By evoking illness and suffering, it can also accentuate the powerlessness of the person, who then becomes the object of care (Richardson, 1999). The impact of wearing a white coat on the relational dynamics between patients or residents (in this case, older individuals) and healthcare professionals has yet to be thoroughly researched. However, certain studies, carried out specifically in nursing homes, can provide some answers.

A study by Charras and Frémontier (2010) aimed to create a home-like atmosphere during meals in a nursing home, and to measure the consequences. To achieve this, the professionals of a small unit (comprising 8 older persons with dementia) decided to share meals with the residents. To achieve their objective, the professionals removed their uniforms during this sociable moment in favour of civilian clothes. After a six-month period, the researchers found a significant increase in the weight of the residents, whereas the control group, which consisted of 10 residents who did not share meals with professionals, experienced a weight loss. In addition, a qualitative analysis of the professionals’ observations revealed an improvement in the quality of interactions (e.g., without the uniform, residents recalled stories from their past more spontaneously and professionals learned about the residents’ lives and preferences).

In another study, Charras and Gzil (2013) compared two specialised units for older adults (with dementia) for three months. In the first group (an experimental group of 13 residents), the professionals dressed in civilian clothes, even outside of meal times. In the second group (control group of 14 residents), the professionals wore their uniforms as usual. After three months, the researchers found a significant increase in the quality of life among residents from the experimental group (measured using the QoL-Alzheimer’s Disease scale). The qualitative data from this study provide relevant insights into the removal of the white coat. One of the things that professionals without uniforms find is that residents engage in conversation more readily (e.g., compliment them on their outfit), and are more likely to communicate to discuss something rather than to ask for help. For their part, professionals without white coats feel closer to the residents and find them more autonomous (e.g., the residents perform tasks such as sweeping and clearing tables more spontaneously). By taking off their white coats, the professionals reduce the interpersonal differences that exist between themselves and the residents, which helps to rebalance the relationship (Charras & Gzil, 2013).

In a recent study (Bailly et al., 2020), professionals in two specialised units for older people with dementia experimented with wearing their usual white coat uniform for three months and civilian clothing for another three months (the order was counterbalanced between the two units). Using cameras placed in the institutions, the researchers were able to film the residents (n = 24) and professionals interacting during teatime. A total of 24 15-minute videos were recorded, of which 12 videos depicted the three-month period in uniform, while 12 videos documented the three-month period in civilian clothing. Based on these video clips, the behaviours of the professionals and residents were recorded and analysed (e.g., eye contact, smiling, rubbing their hands together due to anxiety, etc.), as was the content of the conversations and proximal interactions. Statistical analyses revealed that when professionals were not wearing white coats, they interacted with residents more and anxiety behaviours decreased. The authors also note that conversational content changed (i.e., significantly less care-related content, replaced by more personal content), and a greater closeness in interactions was observed.

In a qualitative experimental study, this time conducted in a hospital setting (Sparrow, 1991), nurses who removed their white coats for a period of two months found that this encouraged them to approach (adult) patients differently. By no longer being directly identifiable by their uniforms, nurses could not imagine, for example, taking a patient’s pulse without first having a conversation with them. According to healthcare professionals themselves, wearing a uniform enables quick access to the patient’s body and treating it as an object of work. This way, the uniform constitutes what could be considered a “pass” to the patient’s body.

By making wearing a uniform optional, Scott (1960) found that he could distinguish between two types of nurses: (a) those who chose to wear the uniform were task-oriented and preferentially worked under medical domination, while (b) those who chose to wear their own clothes, leaning more towards interpersonal relationships, and worked in partnership with the multidisciplinary team (Sparrow, 1991).

More recently, in a psychiatric rehabilitation unit in Hong Kong, professionals were able to share their opinions and observations after experimenting with a non-uniform policy for one year (Chu et al., 2020). Among the most significant effects, healthcare professionals mentioned improved relationships with patients as well as improved relationship between professionals. By wearing civilian clothing, the professionals’ sense of authority decreased, leading them to be more friendly and approachable. In addition, the absence of uniforms reduced self-stigmatisation among users and created a more relaxed, home-like environment. Early studies of uniforms have shown that service users feel less intimidated, exhibit less violent behaviour, and develop more trusting relationships with nurses who do not wear uniforms (Lavender, 1987).

These studies indicate that eliminating the uniform would reduce the relational inequality of “dominant-dominated” or “power-dependence” that would, in certain contexts, be the source of what Richardson (1999) considers a subtle form of violence (e.g., paternalistic and condescending attitudes). The removal of the white coat would promote more authentic, personal, and human interaction between equals. By erasing the ambient medicalisation and medical dominance, we could then observe a humanisation of care and relationships, allowing for the existence of the social body beyond the biological one.

## Discussion

Based on extensive empirical evidence, this narrative review highlights the negative and often hidden impact of wearing the white coat uniform. To the best of our knowledge, there is no review that examines the repercussions of wearing a uniform on the patient (in this case, an older patient), the healthcare professional, and on their relationship.

The WCE studies extensively illustrate the unconscious physiological effects of the white coat and of the medical environment on patients and particularly older patients. The underlying processes are still the subject of debate. It is currently proposed that the white coat and/or the medical environment, following classical conditioning, act as conditioned stimuli and give rise to a conditioned anxiety response (activation of the sympathetic nervous system), which can lead to an increase in blood pressure (Ogedegbe et al., 2008). This stress reaction is also likely to impact the memory performance of older adults, and more specifically of older people with a low sense of memory self-efficacy (Schlemmer & Desrichard, 2018). When we know that the diagnosis of MCI (Mild Cognitive Impairment) is made on the basis of the patient’s concerns and a neuropsychological assessment most often performed in a medical environment, this obviously raises questions. In addition to the question of the relevance of making such a diagnosis (Van der Linden & Juillerat, 2014) the issue of its reliability also arises (Morand et al., 2020).

The theory of “enclothed cognition” provides scientific evidence on the impact of the professional wearing a uniform. Wearing a white coat associated with the abstract concepts of care and help would increase the likelihood of expressing pro-social behaviours (López-Pérez et al., 2016). Although these studies were not conducted in the specific context of geriatric care, we assume that the white coat uniform explains or reinforces the over-helping attitude already observed among healthcare professionals in this sector (Sánchez-Izquierdo et al., 2019). The excessive assistance and paternalistic attitudes of professionals (especially in nursing homes) is particularly problematic as it hinders the autonomy and freedom of older adults (van Loon et al., 2021).

The relational dynamic between the patient and the professional is also impacted by the presence, or absence, of the white coat. The absence of uniforms would establish a relationship of equals and force professionals to humanise their care (Bailly et al., 2020; Charras & Gzil, 2013; Richardson, 1999). The resulting authenticity and relational reciprocity could promote a person-centred care model, which is currently recommended in the geriatric care sector (Kogan et al., 2016).

Moreover, recent scientific data also encourages reconsidering the purely practical aspects of the white coat. The three practical arguments generally put forward to justify the wearing of a uniform are that it: (a) proclaims the identity of the wearer; (b) ensures cleanliness and hygiene; and (c) is comfortable and allows freedom of movement (McLean & Naidoo, 2006; Pearson et al., 2001). With respect to the identification of professionals, empirical evidence suggests that it can easily be achieved through other means, such as wearing a badge (Chu et al., 2020). In the study by Charras and Gzil (2013), residents of an institution (with a neurodegenerative disease) recognised professionals even though they were dressed as civilians. A badge was introduced to help families identify the professional staff. As far as the hygiene criterion is concerned, the white coat can, in fact, carry numerous bacteria, some of which are particularly resistant (Nash, 2014; Treakle et al., 2009). Kumar et al. (2020) found that out of 120 white coats worn by medical staff in a hospital, 73% were contaminated with various bacteria, making the uniform a potential source of infections rather than a protective form of clothing. It is for this reason that, in 2007, the Department of Health in the United Kingdom published a code of recommendations for good dress practices, asking doctors to roll up their sleeves and remove their watches, ties, and white coats (Jacob, 2007). A study carried out in the same country indicates that out of the 86 participating doctors, 70% of them believe that the white coat presents a risk of infection, while 60% find it uncomfortable (Douse, 2004).

Despite these findings, the white coat is omnipresent in geriatric care settings. Beyond its purely practical role, this white coat would take on, in a more covert way, essential symbolic functions for the professionals. In particular, the uniform can create a clear distinction between the patient group (“them”) and the caregiver group (“us”), which can induce a social, psychological and relational distance, which some professionals consider necessary and protective (Pearson et al., 2001; Richardson, 1999). In a qualitative study, Shaw et al. (2010) conducted semi-structured interviews with 14 nursing students to understand the impact of uniforms on self-image and professional identity. The analysis of their answers reveals the importance they place on the uniform due to its symbolic role and identity function. Putting on the uniform allows one to proclaim one’s professional identity, to put on the role of a healthcare professional so that the associated skills are implied. As one study participant explains: *“To me, your uniform reflects the pride you have in your job…you don’t have pride in yourself, you don’t have pride in your job. Therefore, you are not going to be as willing to provide good care”* (participant 10) (Shaw et al., 2010, p. 22).

### Clinical implications: should the white coat be removed?

There is now a large body of robust scientific evidence about the potential negative effects of wearing a white coat in the geriatric care setting. These elements lead us to legitimately question whether or not it is relevant to continue using the uniform, as well as the reasons for upholding this custom and the consequences that a possible change would bring. The removal of the white coat, which can be initiated as the first step in the process of demedicalisation, can be relevant in specific contexts such as the living environments of older adults (nursing homes, homes). Indeed, in these places, the white coat, worn continuously, would hinder the highly recommended person-centred approach (Van der Linden & Juillerat, 2014; Charras & Gzil, 2013). In recent years, numerous studies have demonstrated the value of adopting a culture change within residential facilities, which involves creating a home-like environment (Duan et al., 2020). It is obvious that this change in culture and environment must be accompanied by the removal of the uniform. However, this simple act of removal alone does not constitute a change in the philosophy of support and care. The removal of the white coat could also be favourable in other specific situations, such as during diagnostic cognitive assessments where the medical environment and the white coat can impede the performance of older adults. Although more pronounced among older adults, the white coat effect is not specific to this population. The impact of wearing a white coat on patients, professionals, and their relationships should, therefore, be considered in a global context. The removal of the white coat in the context of long-term hospitalisation (e.g., psychiatric hospitalisation for young people) should also be further investigated and studied.

Beyond the question of where (i.e., in what environment removal of the white coat would be relevant), we believe it is also essential to ask the question of “how?”. We cannot ignore the symbolic function of the white coat for healthcare professionals (manifestation of professional identity and skills; psychological protection), nor the fact that it is currently preferred by older patients, as it represents competence, professionalism, and therefore safety. Removing the white coat could be accompanied by reluctance on the part of professionals and older adults. In this sense, it is essential to consider the most optimal implementation method; the one that generates the least inconvenience to everyone. To our knowledge, there is no scientific study on this subject. The removal of the white coat in specific geriatric sectors such as nursing homes should therefore be investigated in further empirical research that would aim to (1) assess the relevance of this change; (2) identify the effects (positive and negative) that this change implies for the different actors involved (professionals, older people, family members); (3) test various implementation procedures (e.g., gradual vs. instantaneous removal) and methods of use (e.g., never using the white coat vs. using the white coat only for medical acts and not for daily acts).

## Conclusion

The increasingly controversial white coat is ubiquitous in geriatric care. This narrative review is the first to combine the abundant scientific data and several theoretical frameworks to highlight the pitfalls of adopting such a uniform. While it is appropriate in some specific contexts (e.g., emergency and acute settings), we also understand that this simple item of clothing is, through powerful symbols, detrimental to the older patient (“white coat effect”), as well as the healthcare professionals (“enclothed cognition”), and their relationship.

Because it has the power to fix roles, to dehumanise relationships, to “pathologise” behaviours, the wearing of white coats constitutes an obstacle to what is widely recommended today in supporting older adults, i.e., the person-centred approach. The removal of the white coat is part of the process of demedicalisation of the nursing home. This change is one of the measures recommended by Van der Linden and Juillerat Van der Linden in their book “Penser autrement le vieillissement” (2014, chapter 4). Through this narrative synthesis on the white coat, we intend to awaken reflection on the (over)medicalisation of ageing and question, as proposed by Martial Van der Linden, the reductive biomedical prism through which we consider ageing in our current society. To change the way we think about ageing, Martial Van der Linden invites us to see vulnerability as part of our identity rather than as a reason for exclusion. He proposes to cherish the fragility that we all share and makes us human. This reasoning is in line with what Donald Berwick (Harvard physician and former administrator of CMS) said about the white coat during his 2010 Yale Medical School commencement speech: “… To become a healer, you must do something even more difficult than putting your white coat on. You must take your white coat off. You must recover, embrace, and treasure the memory of your shared, frail humanity of the dignity in each and every soul. …” (Pfeifer, 2020).
